# Direct Measurements of the Volume Flow Rate and Emissions in a Large Naturally Ventilated Building

**DOI:** 10.3390/s20216223

**Published:** 2020-10-31

**Authors:** David Janke, Qianying Yi, Lars Thormann, Sabrina Hempel, Barbara Amon, Štěpán Nosek, Philippe van Overbeke, Thomas Amon

**Affiliations:** 1 Department Engineering for Livestock Management, Leibniz Institute for Agricultural Engineering and Bioeconomy (ATB), Max-Eyth-Allee 100, 14469 Potsdam, Germany; QYi@atb-potsdam.de (Q.Y.); lthormann@atb-potsdam.de (L.T.); shempel@atb-potsdam.de (S.H.); tamon@atb-potsdam.de (T.A.); 2 Department Technology Assessment and Substance Cycles, Leibniz Institute for Agricultural Engineering and Bioeconomy (ATB), Max-Eyth-Allee 100, 14469 Potsdam, Germany; bamon@atb-potsdam.de; 3 Faculty of Civil Engineering, Architecture and Environmental Engineering, University of Zielona Góra, Licealna 9/9, 65-417 Zielona Góra, Poland; 4 Institute of Thermomechanics, The Czech Academy of Sciences, v.v.i., Dolejškova 1402/5, 18200 Prague, Czech Republic; nosek@it.cas.cz; 5 Flanders Research Institute for Agriculture, Fisheries and Food (ILVO),Technology and Food Science Unit, 9090 Melle, Belgium; philippe.vanoverbeke@ilvo.vlaanderen.be; 6 Free University Berlin (FUB), Department of Veterinary Medicine, Institute of Animal Hygiene and Environmental Health, Robert-von-Ostertag-Str. 7-13, 14163 Berlin, Germany

**Keywords:** boundary layer wind tunnel, air exchange, laser Doppler anemometer, flame ionization detector, dairy barn

## Abstract

The direct measurement of emissions from naturally ventilated dairy barns is challenging due to their large openings and the turbulent and unsteady airflow at the inlets and outlets. The aim of this study was to quantify the impacts of the number and positions of sensors on the estimation of volume flow rate and emissions. High resolution measurements of a naturally ventilated scaled building model in an atmospheric boundary layer wind tunnel were done. Tracer gas was released inside the model and measured at the outlet area, using a fast flame ionization detector (FFID). Additionally, the normal velocity on the area was measured using laser Doppler anemometry (LDA). In total, for a matrix of 65 × 4 sensor positions, the mean normal velocities and the mean concentrations were measured and used to calculate the volume flow rate and the emissions. This dataset was used as a reference to assess the accuracy while systematically reducing the number of sensors and varying the positions of them. The results showed systematic errors in the emission estimation up to +97%, when measurements of concentration and velocity were done at one constant height. This error could be lowered under 5%, when the concentrations were measured as a vertical composite sample.

## 1. Introduction

Agriculture is a main contributor to the European ammonia and greenhouse gas (GHG) emissions—around 11% for ammonia [1] and 37% for GHG can be related to the livestock management of cattle [2]. Accurate measurements are the basis for efficient emission mitigation measures. Dairy cows are mainly housed in naturally ventilated dairy barns (NVDBs) with large openings, connected directly to the ambient, turbulent weather conditions. This makes the measurements of volume flow rate and gaseous emissions from these buildings challenging. In NVDBs, volume flow rate and gaseous emissions are usually measured by indirect mass balancing methods, wherein the air exchange rates can be derived from measuring the dilution of a tracer gas with a known release rate. The emission rate of the target gas can then be derived as the product of the air exchange rate and the target gas concentration. Further detailed information on mass balancing methods can be found, e.g., in Eren Ozcan et al. [3] and Ogink et al. [4]. Indirect methods are based on the assumption of the perfect mixing of tracer gas and target gas. Since in reality, this assumption can not be fulfilled, high uncertainties in the estimation of emissions can occur [5,6]. If the carbon dioxide (CO_2_) produced by the animals is used as tracer gas, which is the most common approach, additional error sources can occur in the estimation of the CO_2_ source term, which directly biases the estimation of the air exchange rates [7]. These additional error sources can be related to animal parameters, such as weight, activity, productivity and pregnancy, and also additional CO_2_ sources, such as manure [6].

A more straightforward approach to estimate volume flow rate and emissions is the direct method. As the name suggests, in this approach, the velocity vectors of the airflow entering or leaving the NVDB are directly measured and used to calculate the volume flow rate and air exchange rate (a measure of how many times per hour the air within a room is replaced). Additional to the velocity, the associated concentration of target gas in the leaving airflow needs to be measured. The emission rate of the barn can then be computed as the product of directly measured volume flow rate and the simultaneously measured gas concentration.

The challenge in applying direct methods for NVDB is to find representative sensor positions for the measurement of both velocity and concentration. Due to the direct coupling of the large openings with unsteady weather regimes, it is impossible to define constant inlets or outlets [8,9]. Hence, under real conditions, an increasing number of sensors for velocity and gas concentration can be expected to increase the accuracy of the results. Since the costs for sensors are high, usually the aim is to have a most efficient measurement design, meaning the lowest possible number of sensors with the maximum possible accuracy.

De Vogeleer et al. [10] systematically investigated the influence of the density of sampling positions for estimating the volume flow rate in a mock-up test building of a pig barn, with side openings of 0.5 m × 3 m. They also measured the concentrations of a tracer gas at varying positions, but did not use the results for the direct estimation of emissions, but to compute the volume flow rate with an indirect method and compare the results with the direct method. Generally, it was found that better accuracy was obtained with a higher number of sampling locations, and unrepresentative selected sampling locations could lead to systematic overestimation or underestimation of the airflow rates.

So far, no systematic investigation of the influences of the number and positions of sensors on the direct estimation of both air exchange and emissions on a real scale NVDB has been done. This is most likely due to the immense effort needed: in order to measure most accurate results that could act as reference data for large NVDBs, a very high number of sensors for both velocity and concentrations would be needed. Under real conditions, installing these would be highly impractical and expensive.

Wind tunnel measurements can be a promising alternative. Provided that similarity laws are obeyed, arbitrary constant boundary conditions can be applied to scaled down models, and the gained results can be transferred back to the natural scale [11]. For agricultural applications, several studies in recent years have been conducted to investigate animal husbandry systems. Zhang et al. [12] investigated the influences of obstacles upstream of a fully opened pig barn on the transport and dilution of a pollutant gas on the downwind side of the barn. They found that the presence of obstacles could reduce the pollution in the area downstream the barn. Aubrun and Leitl [13] investigated the dispersion of odor around a pig barn in a 1:400 scaled model. By properly modeling the unsteady behavior of the flow, they could identify the plume characteristics as the main effect for dispersion of odor. Several studies investigated the ventilation performances of naturally ventilated barns [14,15,16,17,18,19,20]. Shen et al. [20] investigated a 1:25 scaled model of a NVDB and found that the positions of the openings had no influence on the total air change rates, but the size of the opening did. Yi et al. [16] also investigated the influences of the positions of the openings of a 1:40 scaled model of a NVDB. They found that the opening position had a major impact on the inside flow pattern and on the internal air changes in the animal occupied zones. Nosek et al. [21] investigated the airflow patterns and the dispersion of a tracer gas inside a 1:50 scaled model of a NVDB. They found that the size of the openings and the roughness of the terrain had a significant effect on the airflow pattern and also the dispersion of the tracer gas. De Paepe et al. [18] and De Paepe et al. [17] investigated a 1:60 scaled model of a NVDB. They found that with a fully open outlet, the inside velocities were up to four times higher [18], and that the total air change rates were affected by the inflow angle [17].

These studies illustrate the benefits of the method wind tunnel modeling, which allows measurements in high detail, both for velocities and gas concentrations, and under reproducible and stable boundary conditions. However, until now, the direct measurement of emissions from a NVDB (by measuring both the gas concentrations and velocity vectors at the openings of the NVDB) was not yet investigated with this method.

In this study, we applied high resolution wind tunnel measurements to derive a reference dataset for both volume flow rate and emissions of a NVDB. Based on this dataset, variations of sampling configurations with a systematic reduction of the number of sensors were investigated and assessed regarding the resulting accuracy. We hypothesized that: (1) The positioning of the sensors introduces a systematic bias to the results, which is caused by an in-homogeneous velocity distribution and an imperfect mixing of the gas concentrations. (2) The spatial variation of the gas concentrations introduces a larger systematic error due to sensor positioning than the spatial variation of velocity. The aims of this study were to (1) quantify the impacts of number and position of sensors on the estimations of volume flow rate and emissions and (2) to derive practical advice for measurement setups in terms of number and positioning of these sensors.

This study investigated the most basic scenario of flow through a NVDB and is considered as the prelude for following campaigns with incrementally increased complexity of flow and building geometry. Hence, following simplifications in the modeling and measurement setup were made:

(a) The barn was investigated under straight cross-flow, isothermal conditions and no variation in the inflow direction. (b) The side-openings were fully open and did not change size or position. (c) The presence of animals inside the barn was not modeled. (d) Only the advective pollution flux was measured, not the turbulent pollution flux. The potential implications of the respective simplifications (a)–(d) are discussed in the Results and Discussion sections.

## 2. Materials and Methods

### 2.1. Wind Tunnel

**Figure 1:**
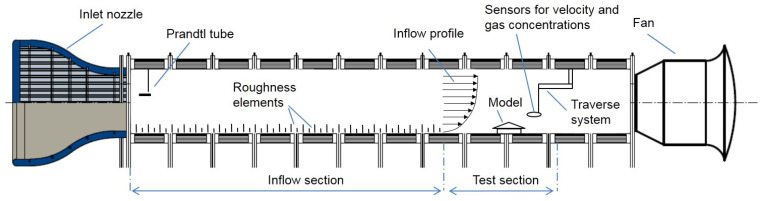
Sketch of the atmospheric boundary layer wind tunnel at ATB.

The experiments were conducted in an atmospheric boundary layer wind tunnel (ABLWT) at ATB Potsdam; see Figure 1.

The ABLWT was specially designed to investigate ventilation and dispersion processes in agricultural buildings [15,16,22]. It is 28.5 m long, consisting of an air inlet fitted of honey combs, an air outlet equipped with an axial fan and a 19.5 m long inflow section to develop the atmospheric boundary layer. The cross-sectional area of the test section had a width of 3 m and a height of 2.3 m.

### 2.2. Studied Building and Gas Release

The studied building is a naturally ventilated dairy barn located in Northern Germany, with a building envelope typical for that region. The barn has a length of 96 m and a width of 34 m, and the roof height varies from 4.2 m at the side walls up to 11.4 m at the gable top. The barn has the capacity for 375 dairy cows, with completely open side walls and a ridge opening with a width of around 0.5 m. During frost periods, the opening of the side walls is reduced or completely closed with wind screens. Further detailed information about the barn can be found in Konig et al. [7].

**Figure 2:**
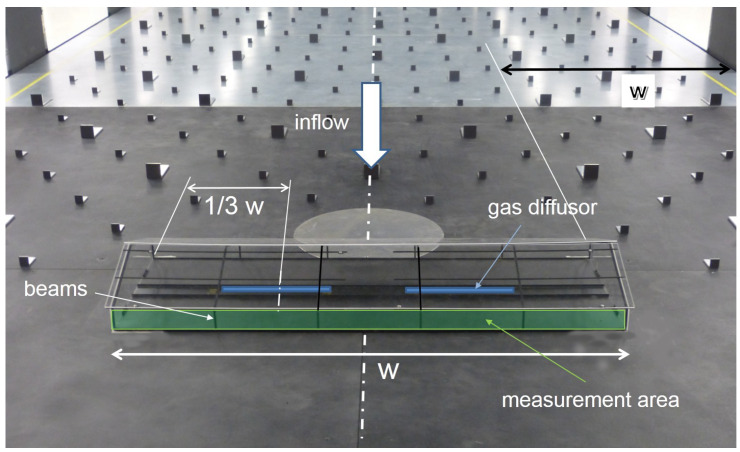
Scaled model positioned inside the wind tunnel. Gas was released with two gas diffusion stones, marked blue. Normal velocities and gas concentrations were measured at the green marked outlet plane.

A 1:100 scaled model of this building was made of 2 mm thick acrylic glass and a supporting structure, shown in Figure 2.

The model was built with closed gable walls and a closed ridge opening, but completely opened side walls. The floor geometry with an elevated feeding alley and entrance edges was modeled, but the presence of animals was not modeled, since a previous study indicated that their effect was insignificant to the flow pattern and pollutant transport under cross-flow conditions [21]. The model was investigated in Janke et al. [23], where more detailed information about the geometry can be found.

The model was placed at the symmetry line of the wind tunnel, oriented with sidewall openings perpendicular to the approaching flow and the measurement area at the downwind side. The blockage ratio of the scaled model to the cross-section of the wind tunnel was 1.6%, which is less than the recommended maximum value of 5% for wind tunnel tests in VDI-guideline 3783/12 [11], and thus the tunnel effect can be neglected. Figure 2 shows the wind tunnel with the scaled model placed inside.

**Figure 3:**
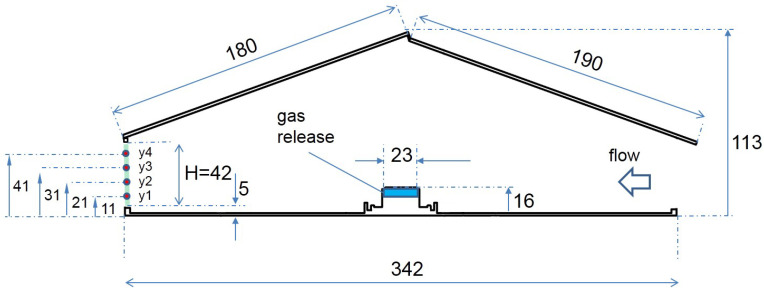
Sectional view of the scaled model. Dimensions are in mm. The green line at the outlet opening marks the measurement area.

Along the longitudinal symmetry line of the model, two gas diffusers were mounted on the floor. They consisted of porous stone material (Marina Extendable Airstone, HAGEN GROUP, Holm, Germany). They had a height of 13 mm, a width of 23 mm (shown in Figure 3) and a length of 260 mm and were symmetrically positioned with their middle point at 1/3 w and 2/3 w, according to Figure 2.

The height of the gas release would be 1.3 m in real scale and was chosen to mimic the animals’ mouths heights, where most of the CO_2_ and also methane is released. The diffusors were positioned along the feeding alley of the real scale barn, so the scenery of eating cows was simulated. The positioning of the source is further discussed in the discussion part. The gas used in this study was ethane. A controlled flow rate of 120 std l h^−1^ (standard liters per hour) of a gas mixture consisting of 50% ethane and 50% synthetic air was realized with mass flow controllers (red-y smart controller GSC, Vögtlin Instruments AG, Switzerland). The gas mixture was injected into the two gas diffusers with a symmetric tube system. Preliminary studies were done to ensure uniform gas release along the length of each diffuser. With the overall surface area of the diffusers of 0.01196 m^2^, the given volume flow of 120 std l h^−1^ under ambient conditions with 20 °C resulted in an average vertical exhaust tracer velocity w_*t*_ of 0.003 m/s, so that the flow field was not disturbed by the momentum of the injected tracer gas. The gas mixture was chosen after preliminary tests in the outlet area, in order to gain a sufficient signal-to-noise ratio with the fast flame ionization detector (FFID).

### 2.3. Sensors and Additional Equipment

Air velocities at the measurement area were measured using a 2D fibre-optic laser doppler anemometer (LDA) (Dantec Dynamics, Skovlunde, Denmark) combined with the BSA Flow Software package (Dantec Dynamics, Skovlunde, Denmark). The LDA probe head was 0.06 m in diameter and 0.45 m in length and provided a focal length of 0.25 m. The precision of the LDA measurements for the velocities in flow direction was determined with repeated measurements of a vertical velocity profile and found to be <1%.

Gas concentrations were measured using a fast-flame-ionization-detector (FFID) (HFR400 Fast Response FID, Cambustion, Cambridge, England). The gas was sampled with a sampling needle 200 mm long with a diameter of 0.24 mm. Before the measurements, the FFID was calibrated with calibration gas (ethane) of three different concentrations (300 ppm, 1000 ppm, 5000 ppm). The measured mean concentrations at the outlet area were in a range of 5 ppm and 850 ppm. The test gas was also used to derive the uncertainty of the FFID, which was found to be below 1%.

Both the LDA probe and the FFID probe were mounted one after another on a three-dimensional computer-controlled traverse system that allowed automated and precise probe positioning with an uncertainty of <0.1 mm. A fog generator Tour Hazer II (Smoke Factory, Burgwedel, Germany) was placed at the wind tunnel inlet to produce seeding particles for LDA measurements.

The free stream wind speed at the wind tunnel inlet was measured using a Prandtl tube, connected to a pressure transducer MKS Baratron® Type 120A (MKS Instruments, Andover, MA, USA). The Prandtl tube was located at the center of the entrance of the test section at a height of 1.3 m from the wind tunnel floor.

### 2.4. Measurement Procedure

**Figure 4:**
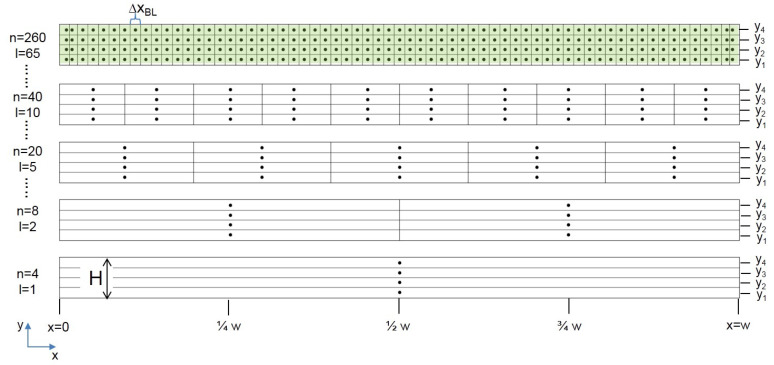
Lateral (x-direction) and vertical (y-direction) division of sampling positions. l is the number of vertical divisions and is increased incrementally from 1 to 65. n is the resulting total number of sampling points. The green marked area is the baseline configuration with the maximum amount of sampling points, with $\Delta x_{BL}$ being the width of the area around each sampling point.

Measurements were done at the outlet area of the model shown in Figure 2. A matrix of 65 sampling points (SPs) in x-direction times 4 SPs in y-direction was measured, resulting in 260 SPs overall, sketched in Figure 4.

**Figure 5:**
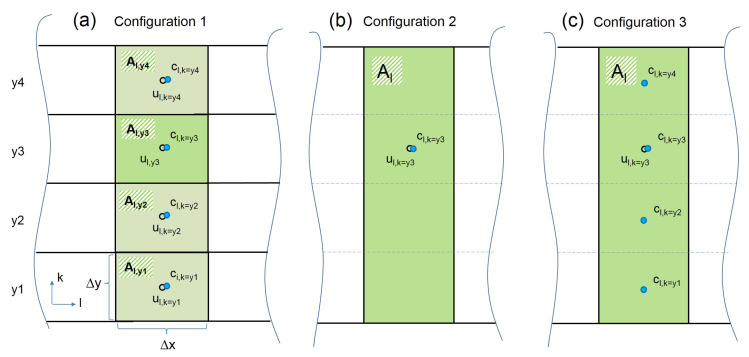
Construction of the area for each sampling point and the calculation of volume flow rates and emissions with the three configurations. The index *l* is for the lateral division of the measurement area in x-direction ([1...65]), the index *k* is the vertical position ([y1, y2, y3, y4]). Black dots and blue dots symbolize measurements of the velocity *u* and gas concentration *c*, respectively. Subfigures (**a**), (**b**), and (**c**) show configurations 1, 2, and 3, respectively. For configuration 1, the emission rate for a lateral division *l* is computed as $E_{l}=\sum_{k=1}^{4}A_{l,k}\cdot u_{l,k}\cdot c_{l,k}$. For configuration 2, the emission rate for a lateral distribution *l* and a vertical position k (in this example, k = y3) is computed as $E_{l,y3}=A_{l}\cdot u_{l,y3}\cdot c_{l,y3}$. For configuration 3, the emission rate for a lateral distribution *l* and a vertical position k (in this example, k = y3) is computed as $E_{l,y3}=A_{l}\cdot u_{l,y3}\cdot \frac{1}{4}\left(c_{l,y1}+c_{l,y2}+c_{l,y3}+c_{l,y4}\right)$.

In the following, this set of SPs will be called *baseline*. The width of the area around each sampling point for the baseline was $\Delta x_{BL}$= 15 mm, which corresponds to 1.5 m in the real scale. The four vertical SP positions were at heights of 11 mm for y1, 21 mm for y2, 31 mm for y3 and 41 mm for y4, shown in Figure 3. This resulted in a height of the area around each sampling point (marked as Δy in Figure 5) of 11 mm for SPs on y1 and y4 (1.1 m in real scale), and 10 mm for SPs on y2 and y3 (1.0 m in real scale).

### 2.5. Boundary Layer

The total height H of the outlet area was 42 mm; the total width W was 966 mm.

First, the LDA probe was mounted on the traverse system and velocities were measured. A sample number of 60,000 at the outlet was found to reach statistically converged results. Hence, every sample location was measured until this sample number was reached. The sampling rate varied between approximately 90 and 500 Hz, depending on the sampling position and concentration of seeding particles.

After velocity measurements were completed, the FFID probe was mounted on the traverse system and the same measurement positions as with the LDA were measured. Measurements of the gas concentrations at each point were done with a sampling frequency of 500 Hz. After preliminary tests at three different sampling heights on the outlet area, a sample number of 20,000 was found to deliver statistically converged results of the mean concentration values; hence, each sampling point was measured until this sample number was reached.

### 2.6. Investigated Configurations

Variations of three different configurations were investigated. Configuration 1 took into account all vertical sampling positions y1, y2, y3 and y4 at each lateral sampling division, sketched in Figure 5a).

For real scale measurements, sensors are usually not positioned on top of each other at several heights, but distributed along one constant height [8,9]. This is reflected in configuration 2, where only one sampling point at the height of y1, y2, y3 or y4 was chosen for each lateral division. The respective area for the sampling point was computed as related width of the sampling point (same as in the first variation) multiplied by the height H of the measurement area; see Figure 5b).

Configuration 3 combines configurations 1 and 2. Volume flow rates were computed the same way as in configuration 2 with only one constant height. However, for real scale measurements, gas concentrations are often not measured with single sampling points but as spatial composite samples; sampling tubes with several openings are used. Usually, the spatial composite sampling is done inside the barn at a constant height in lateral directions. This idea was adapted for the vertical direction in configuration 3, where the concentration value was computed as the mean value of the concentrations measured at y1, y2, y3 and y4 at each lateral position, sketched in Figure 5.

All three configurations were varied by incrementally decreasing the number of lateral sampling positions; Figure 4 gives an example for configuration 1.

**Figure 6:**
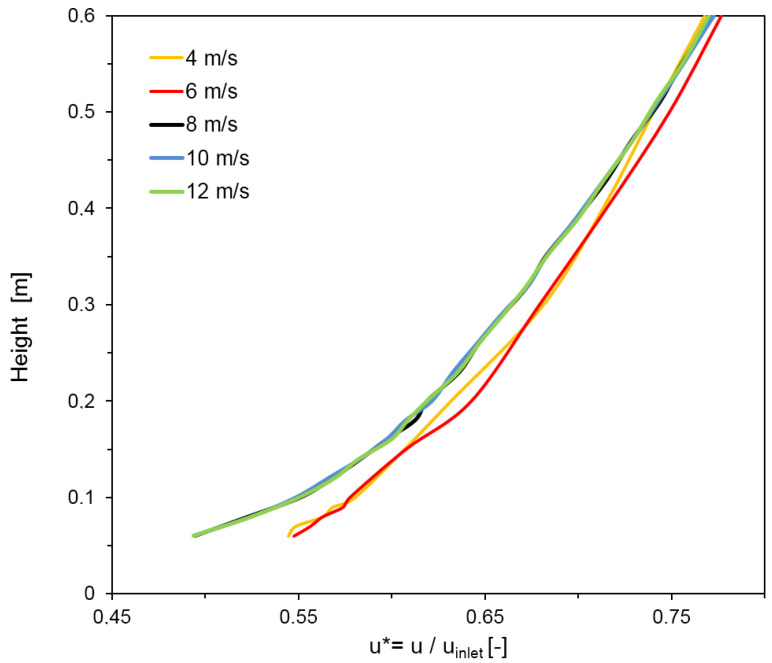
Vertical velocity profiles of the atmospheric boundary layer, with measured velocities u in position on the model. The undisturbed inlet velocity u${}_{inlet}$ at the inlet of the wind tunnel was gradually increased from 4 to 12 m s^−1^. The dimensionless velocity u* was normalized with u${}_{inlet}$. For velocities u${}_{inlet}$ ≥ 8 m s^−1^, u* is constant, meaning that a fully turbulent, and hence Reynolds number-independent boundary layer, is achieved.

Within the wind tunnel, a fully developed turbulent flow with a logarithmic vertical velocity profile was generated though the presence of roughness elements at the inflow section. The vertical velocity profile of the generated boundary layer is shown in Figure 6.

The vertical velocity profile was measured without the presence of the model at the symmetry line of the wind tunnel (dashed white line in Figure 2 at the position, where the inflow opening of the model would be). Reynolds number independence was tested for the undisturbed inflow profile upstream in the model and at the outlet section of the model. The velocity profile was found to be independent of the Reynolds number, when the undisturbed inlet velocity was ≥8 m s^−1^, which is a basic prerequisite for transferring the wind tunnel results of the boundary layer to natural conditions. The inflow profile fulfilled the criteria for a boundary layer over a moderately rough terrain according to VDI [11].

Choosing as characteristic length scale L=0.11 m (approximate height of the barn) and as characteristic velocity scale $u=5\;m\;s^{-1}$ (velocity at the opening of the barn model with an undisturbed wind tunnel inlet velocity of $u=8\;m\;s^{-1}$), and with $\nu =1.48\;\times 10^{-5}\;m\;s^{-1}$ as the kinematic viscosity of air at 15 °C, the internal Reynolds number of the flow is given by

Re=Luν≈37,200

This number fulfills the criterion for an internal independence of the Reynolds number which must be greater than 2 × 10^4^, as stated in Cermak et al. [24]; hence, the inside flow pattern is considered as fully turbulent and transferable to the real scale.

### 2.7. Computation of Volume Flow Rate and Emissions

Gas emissions E were computed following this equation:

(1) $$E=\sum_{i=1}^{N_{SP}}Q_{i}\cdot c_{i}$$

where $N_{SP}$ is the number of sampling points used, *Q* is the volume flow rate and *c* is the gas concentration in the respective sampling point. *Q* and *c* are the mean values of the consecutively measured volume flow and gas concentrations; hence the emissions estimated and presented in this study are the advective mean emission fluxes, but the instantaneous, turbulent emission fluxes were not recorded due to limitations in the setup of the measurement devices. The volume flow rate at each sampling point was computed as the product of the area around the point and the normal velocity vector u pointing out of that area:

(2) $$Q_{i}=A_{i}\cdot u_{i}$$

where $A_{i}$ was constructed dependent on the respective configurations of investigated sampling points, sketched in Figure 5.

### 2.8. Calculation of the Deviations

The results of the measurements for the different configurations are shown in the following section. Deviations are presented as relative errors compared to the baseline dataset with the highest number of measurement points:

(3) $$\Delta_{P}=\frac{P_{CFG}-P_{BL}}{P_{BL}}\cdot 100$$

where the *P* is a placeholder for the considered property (velocity *u* and gas concentration *c*), the subscript *CFG* stands for the investigated sampling configuration and *BL* stands for baseline configuration.

## 3. Results

### 3.1. Baseline Configuration

**Figure 7:**
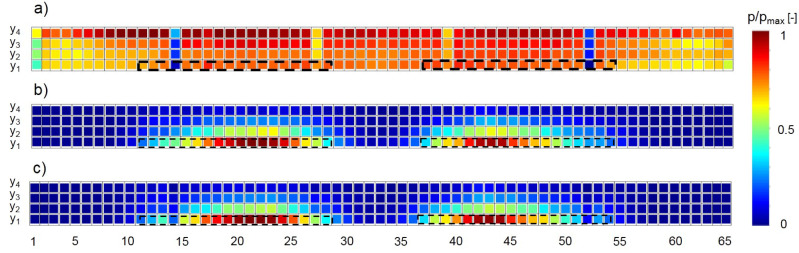
Measured concentrations, normal velocities and calculated emissions of the baseline configuration. Upper (**a**) shows the mean velocity u in normal direction for each sampling point; (**b**) shows the mean concentration c for each sampling point. (**c**) The computed emissions E = Q·c, where Q was calculated as the product of u and the area A that surrounded each sampling point. The results are normalized with the respective maximum values of u, c and E. “p” at the colorbar is a placeholder for the property shown—concentration, velocity or emissions. Numbers on the x-axis index the lateral sampling position. The black dotted lines sketch the positions of the gas diffusers inside the barn.

Figure 7 shows the results for the normal velocity *u* and the gas concentrations measured at the outlet of the baseline configuration.

The values for *u* are spatially dependent. In vertical direction, a trend is visible with higher velocities towards the roof, meaning that the highest velocities were measured at line y4.

In lateral directions, the velocity is accelerated towards the symmetry line of the measurement area, with respective lower velocities in the sampling positions between 1–14 and 52–65. This is probably due to the frictional resistance of the closed sidewalls of the model. A region with lower velocities can be seen at the symmetry line at positions 32 and 33, especially in the lower region. The cause for this is probably the formation of interacting vortex systems of the both gas diffusers, resulting in a deceleration of the flow.

Reduced velocities at sampling positions 14 and 52, and also at positions 27 and 39, can be seen. In the vicinity of these positions, vertical beams of the model’s supporting structure were present; see Figure 2. Although the beams were very thin (2 mm), and measurements were not conducted directly behind them, but with a distance of approximately 1 mm, their influence is still clearly visible.

The values for the gas concentrations c show a heterogeneous distribution of gas concentration at the outlet with a clear accumulation of concentration downstream the two gas diffusers inside the barn. The highest concentrations can be seen on the height of y1 around the symmetry axis of each diffuser. In this area, the highest vertical gradients of concentrations can be noticed, which are at lateral sampling position 21, with 14 times higher concentrations at y1 than at y4. On average, sampling height y1 had seven times higher concentrations than y4. This clearly indicates that the assumption of a well mixed gas is not fulfilled and most of the gas released at the surface of the diffuser was transported towards the ground in the downwind direction.

### 3.2. Volume Flow Rates Estimated with Configurations 1 and 2

**Figure 8:**
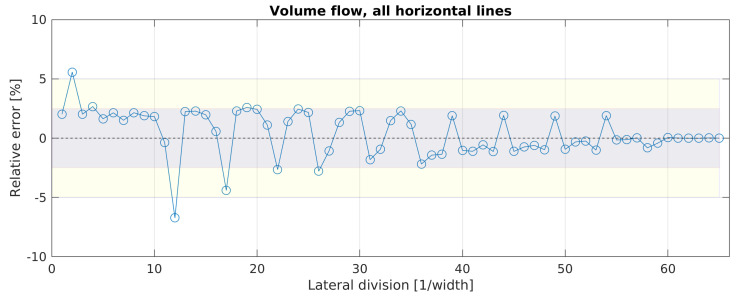
Evolution of the error for estimating the volume flow rate with a growing number of lateral sampling positions, using configuration 1. The dotted line represents the converged mean value (CMV) of the estimated volume flow rate with the maximum number of lateral divisions. The gray and yellow areas mark the error spans of ± 2.5% and ± 5% around the CMV, respectively.

In this section, the results for the volume flow rates estimated with configurations 1 and 2 are presented. As configuration 3 computes the volume flow rate the same way as configuration 2, it is not shown here. For configuration 1, Figure 8 shows the progress of the error for the total volume flow rate estimation with an increasing number of lateral sampling positions.

The magnitude of the error exceeds 5% only for a lateral division of l = 2 and l = 12; for all other divisions, it is less than 5%, where the majority (five exceptions) has an error even less than 2.5%. It should be mentioned that the accurate results with an error <2.5% for only one vertical set of sensors (l = 1) were achieved rather by coincidence. The position of the sensor set l = 1 is on the vertical symmetry line. At this position, the volume flow of the l = 1 set is very close to the average volume flow of all sampling points (baseline configuration). If l = 1 would have been positioned elsewhere, e.g., behind one of the diffusors or near the construction beams, the error would have been greater.

The negative outlier for l = 12 can be explained by the geometrical division. With the configuration l = 12, the lateral sampling positions 14 and 52 were taken into account. As described before and seen in Figure 7, these are the two positions with the lowest velocities in all four vertical sampling points due to the presence of the construction beams. These values decrease the mean value for the estimated total volume flow rate. This behavior can also be noticed for configurations where either lateral sampling positions 14 and 52 or (less effective) 27 and 39 were used, e.g., for l = 17, l = 22 or l = 25. The positive outlier for l = 2 can be explained by the positioning of the two lateral divisions at the symmetry line of each gas diffuser, where the highest velocities occurred, as described in the previous section.

**Figure 9:**
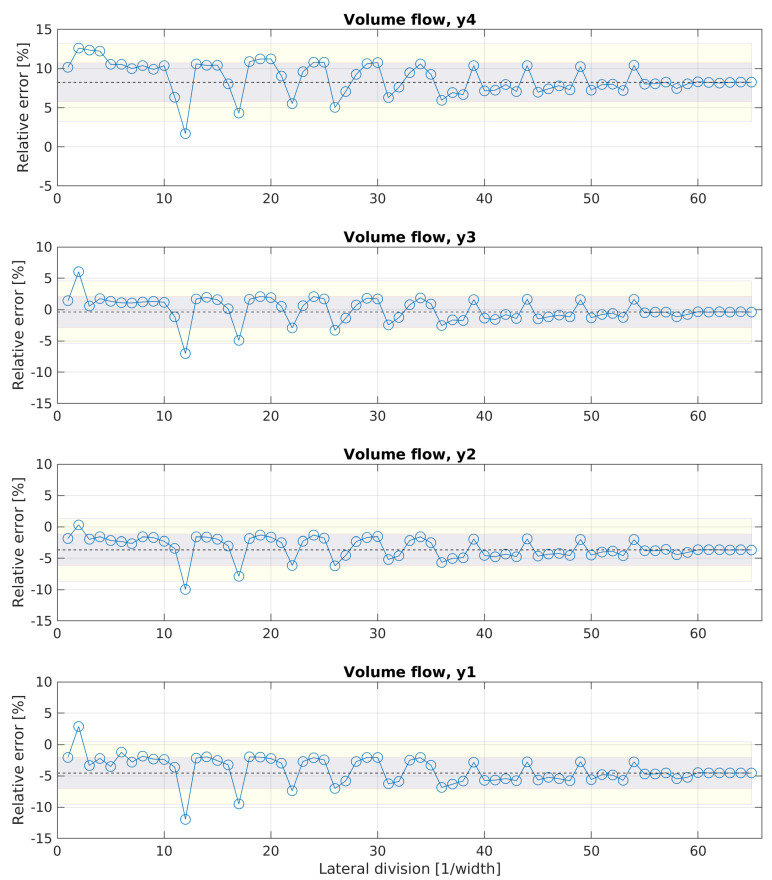
Evolution of the error for estimating the volume flow rate with a growing number of lateral sampling positions and only one constant height of the sampling points. The dotted line represents the value of the estimated volume flow rate with the maximum number of lateral divisions at the respective height.

For configuration 2, Figure 9 shows the progress of the error independently of the lateral division, when sensors would be positioned only on one height.

For all four heights, the error scatters around the converged mean value (CMV), which is estimated with the maximum number of lateral divisions, in a range of ± 5%. At each height, the positive outlier at l = 2 and the negative outlier at l = 12 can be observed, similarly to the characteristic in configuration 1. For all heights except y4, an error span around the CMV less than ± 2.5% was reached with l = 3. However, the values of the CMV can be interpreted as the induced bias of the configuration. The CMV showed a trend towards higher volume flow rates with a rising height. Positioning sensors only at height y1, y2, y3 or y4 would result in a bias of −5%, −4%, ±0% or +8%, respectively, when using the maximum lateral division of l = 65. For the lateral division of l = 3, the bias would be −3.4%, −2%, +0.5% or +12.3%, for y1, y2, y3 or y4, respectively.

### 3.3. Emissions Estimated with Configurations 1, 2 and 3

**Figure 10:**
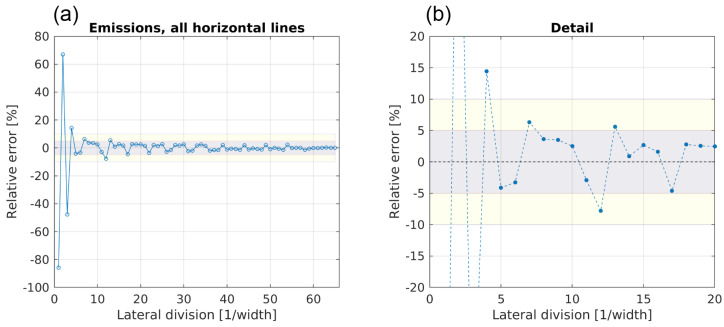
Evolution of the error for estimating the emissions with a growing number of lateral sampling positions, using configuration 1. Subfigure (**a**) shows the error for all 65 lateral divisions, subfigure (**b**) shows a detailed view for the first 20 lateral divisions. The dotted line represents the converged mean value (CMV) of the estimated volume flow rate with the maximum number of lateral divisions. The gray and yellow areas mark the error spans of ±5% and ±10% around the CMV, respectively.

Figure 10 shows the progression of the error for emissions estimated following Equation (1) when using configuration 1.

Compared to the results for the volume flow rate, a wider spread of the error around the CMV is visible, especially until the number of divisions reaches l = 5. The errors for using a lateral division of l = 1, 2, 3 or 4 are −86%, +67%, −48% and +14%, respectively. Beginning with l = 5, the error converges within ±5%, and for l > 30, the error is within ±2.5%. Again, for l = 12, the negative outlier is seen, resulting from measuring low velocities near the construction beams.

**Figure 11:**
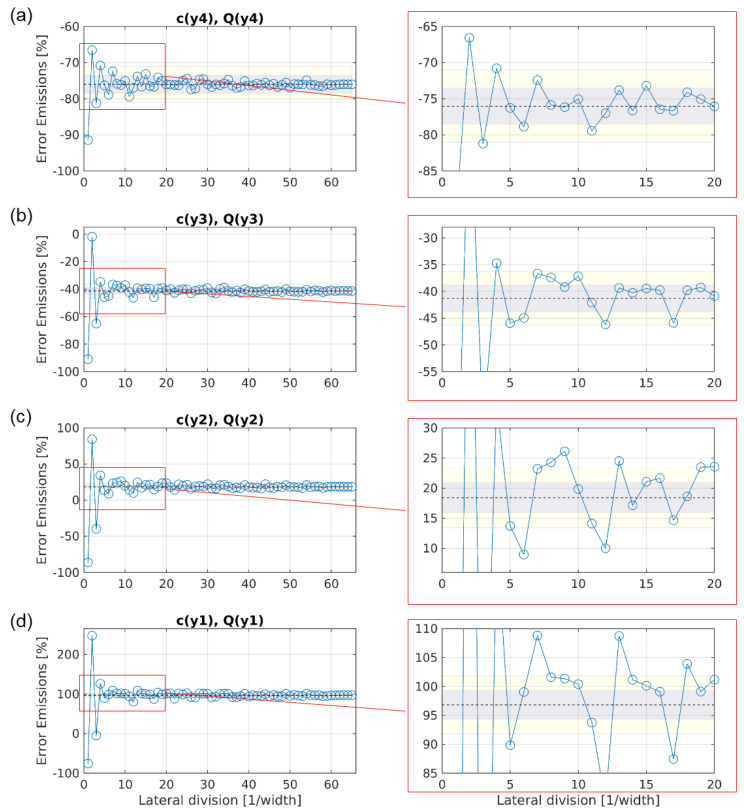
Evolution of the error for estimating the emissions with growing number of lateral sampling positions, using configuration 2. The subfigures (**a**), (**b**), (**c**), and (**d**) show the converging error, when velocities and gas concentrations were only measured at height y_4_, y_3_, y_2_, and y_1_, respectively. The dotted line represents the converged mean value (CMV) of the estimated emissions with the maximum number of lateral divisions. The gray and yellow areas mark the error spans of ±2.5% and ±5% around the CMV, respectively.

Figure 11 shows the progression of the error for emission estimations when using configuration 2.

The spread around the CMV for y4 is similar as for configuration 1, with a converging error within the ±5% area for lateral divisions of l ≥ 3. For y3, y2 and y1, the span of the error around their respective CMV got larger, the nearer to the ground the vertical position was. Accordingly, the number of lateral divisions needed for a converging error within ±5% grew, with l ≥ 5 for y3, l ≥ 13 for y2 and l ≥ 23 for y1. This is a direct reflection of the results for the baseline shown in Figure 7: for y4, the lateral distribution of gas concentration is very homogeneous; the distribution gets more and more heterogeneous the lower the height is. The reason for that is the accumulation of concentration in the vicinity of the gas diffusers. Similarly to the estimated volume flow rate with configuration 2, the CMV show a systematic error, but in contrast to the volume flow rate, the trend was towards lower emission estimations with a rising height. Hence, positioning sensors only at height y1, y2, y3 or y4 would result in biases of +97%, +18%, −41% or −76%, respectively, when using the maximum lateral division of l = 65.

**Figure 12:**
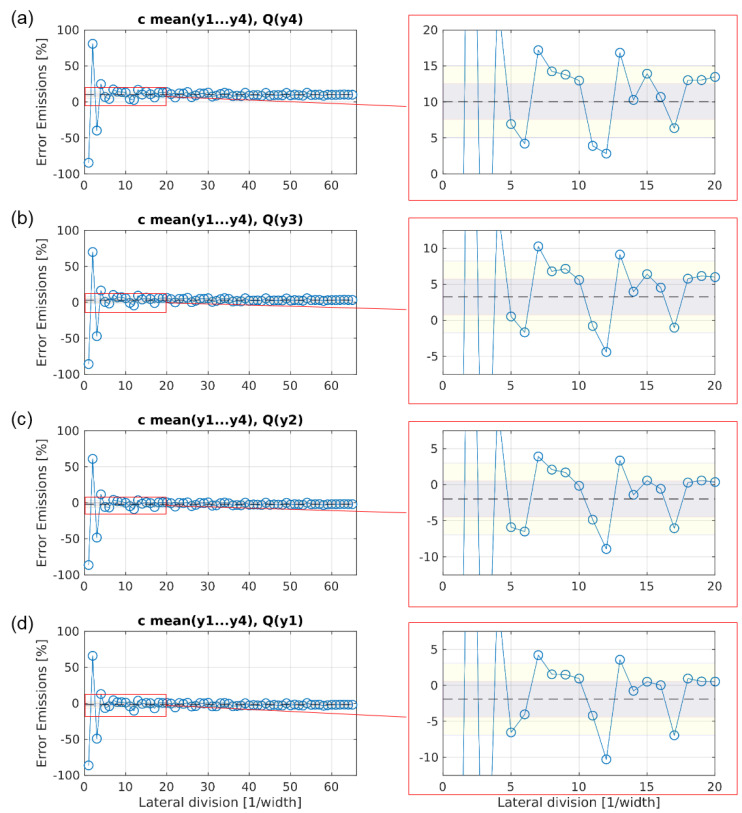
Evolution of the error for estimating the emissions with growing number of lateral sampling positions, using configuration 3. The subfigures (**a**), (**b**), (**c**), and (**d**) show the converging error, when velocities were only measured at height y_4_, y_3_, y_2_, and y_1_, respectively, while the concentration was measured as the mean value of the four vertical sampling points.The dotted line represents the converged mean value (CMV) of the estimated emissions with the maximum number of lateral divisions. The gray and yellow areas mark the error spans of ± 2.5% and ± 5% around the CMV, respectively.

Figure 12 shows the progression of the error for emission estimations when using configuration 3.

The spread around the respective CMV, in contrast to configuration 2, is now for all four heights in the same range with a converging error within the ±5% area for lateral divisions of l ≥ 5. Exceptions to this are recurring outliers for all height at l = 7 (around +6%) and l = 12 (around −7.5%). Compared to configuration 2, the bias for each height has decreased clearly: measuring with configuration 3 the maximum number of lateral divisions l = 65 would result in a bias for the estimated emissions of −2%, −2%, +3% or +10% for y1, y2, y3 or y4, respectively. Reducing the number of lateral divisions down to l = 5 results in a bias of −6.5%, −6%, +0.5% or +7% for y1, y2, y3 or y4, respectively.

## 4. Discussion

The results shown for the baseline indicate that hypothesis (1) can be considered true. Both velocities and concentrations vary spatially in vertical and lateral directions. That biases the results for the estimations of volume flow rate and emissions, depending on the number and positions of the sensors.

Vertical gradients in the velocity at the opening were also reported by De Vogeleer et al. [10] and Van Overbeke et al. [25]. They found a velocity maximum at the center of the opening. In contrast, our results show a maximum towards the top of the opening. This difference can be explained by the smaller opening dimensions typical for a pig barn, with stronger influences from the boundaries and the formation of a parabolic velocity profile. Previous wind tunnel studies with the same scaled model but without the gas diffusers installed showed a nearly constant profile at the outlet [23]; hence we assumed the acceleration towards higher positions was due to the presence of flow obstacles inside the barn. Formations of more or less complex flow patterns due to obstacles inside the barn and their effects on the velocity gradients on the measurement area are hard to predict. Therefore, preliminary tests on the vertical and horizontal velocity gradients can minimize the risk of systematic overestimation or underestimation of the volume flow rate, as was also stated by De Vogeleer et al. [10].

For the volume flow rate, the lateral positioning can introduce negative (−7%) or positive (+6%) biases to the estimation of volume flow rate when using configuration 1. The negative bias can easily be prevented by making sure that sensors are not positioned in the direct vicinity of obstacles, e.g., construction elements. The positive bias is related to less obvious flow disturbances originating inside the barn. In this case, the bias can be prevented by increasing the number of lateral divisions. For configuration 1, a number of l = 3 is sufficient to reach results with errors less than 2.5%. When further increasing l, the results do not improve significantly. Scaling up to the real world, this would mean a lateral distance of 32 m between each sensor. Our simplification (a) (see introduction) of a constant cross-flow might have influenced these results. Under real conditions with varying wind directions, the lateral distances needed would probably decrease, which should be the focus of further research. Still, for configuration 1, a number of l = 3 means a total number of velocity sensors of n = 12, which is a lot if we consider that only one opening of the barn was measured.

When reducing the number of sensors by positioning them laterally distributed only at one height, which is the common approach under practical conditions, a systematic error occurred, as assumed in hypothesis (1). For l = 3, the maximum errors occurred when measuring at either the lowest (−3.5%) or highest (+12.3%) sampling position. When measuring in the vicinity of the horizontal symmetry line of the opening (either y2 or y3), the errors for estimating the total volume flow rate were both below 2%. This proves the common practice of positioning anemometers only at one height (e.g., Joo et al. [8], Wang et al. [9]) to be reasonable, provided the height chosen is reasonable. It also implies that the chance of systematically overestimating or underestimating the volume flow rate is more sensitive to the vertical positioning than to the lateral positioning, provided the minimum lateral distribution (l = 3 in this case) is applied. When these findings should be transferred to the real scale, possible effects of simplification (b) (see introduction) should be considered: When barns with adaptive opening sizes (e.g., screens rolling up and down) are measured, it will be hard to find the optimum sampling height for each screen position. Further research regarding simplification (b) is, therefore, needed.

In this study, the most influential factor on the accuracy of emission estimation is the positioning height of the sensors. If sensors for velocity and gas concentrations are all positioned at one constant height, even with the maximum lateral resolution, the best case is an error of 18%; the worst case is an error of 97%. This is one order of magnitude higher than the errors observed for the volume flow rate; hence we can consider hypothesis (2) true. The reason for the high errors in the different vertical sampling positions is the not-well mixed gas at the outlet, leading to high concentration gradients. This in-homogeneous distribution of gas concentrations was also reported by König et al. [7] for CO_2_, by Wang et al. [26] for NH_3_ and for tracer gas in a wind tunnel study by Nosek et al. [21]. An easy way to smooth these gradients is to apply a sampling system for the concentration measurement device that takes vertical composite samples at each lateral position. By that, the errors could be reduced down to a minimum of −2% and a maximum error of +10% with the maximum lateral distribution. When the lateral number of sensors is further decreased, symmetrical divisions of the width with l ≥ 5 lead to errors between −7 and +4% for (height of 3/8 H), which can be interpreted as a good cost–benefit ratio. On a real scale, this would mean a lateral distance of 19 m between each sensor for the measured NVDB. This number is already close to the minimum lateral distance of 10 m for the sampling of gas concentrations recommended in the VERA test protocol [27]. Just like for the volume flow rate estimations, the number of minimum lateral divisions will probably increase under changing inflow directions and will be the focus of further investigations.

Consequently, in order to reduce the number of sensors for direct measurements of gaseous emissions, the focus should be on an elaborate gas sampling system, which can help to reduce the number of velocity sensors. Spatial distribution of gas sampling can easily be enhanced at on-farm measurements when using, e.g., a tube sampling system with critical orifices. When using open path lasers for the measurement of concentrations at the outlet, one is usually restricted to one constant height. Then, preliminary studies with varying heights could be conducted to quantify the vertical gradients in the gas concentrations and derive height correction factors.

The results shown here were derived for measurements under a stable orthogonal inflow. Although most of NVDBs are aligned towards to a prevalence main wind direction, it can be expected that for real scale conditions, the amount of sensors needed will change for deviating or rapidly changing wind directions. This should be investigated further in future experiments, where the measurements are repeated under varying incident angles.

The investigated dairy barn had the dimensions and a building envelope that are representative for NVDBs in the northern and eastern regions of Germany. The results of this study are expected to be transferable to NVBDs with similar properties, meaning relatively large buildings (>3000 m^2^ floor area) with rather high opening ratios of the side walls (up to 90%) and a focus on cross ventilation. However, for other systems (like rather small barns with small opening ratios, extremely open barns typical for parts of southern Europe, or barns with mainly ridge ventilation), additional experiments should be performed to investigate the influences of the respective building variations on the distribution of velocity and gas concentrations at the opening areas.

Gas concentrations and normal velocities were measured consecutively and their respective mean values were used to estimate the mean emission fluxes (simplification (d)). Depending on the flow scenario, turbulent fluxes, which could not be measured in this study, might have a significant impact on the results, as described, e.g., in Nosek et al. [28]. Hence, further experiments should measure concentrations and velocities simultaneously to quantify the turbulent portion of the emissions.

## 5. Conclusions

Under the simplifications made of a NVDB under constant, isothermal cross-flow with constantly fully-opened sidewalls, the following conclusions can be drawn for the direct measurement of the volume flow rates and emissions:

1. A division of the total opening width into units with a lateral distance of 32 m, each equipped with an anemometer of a height near the horizontal symmetry line, is sufficient to reach results for the volume flow rate with errors less than 5%, compared to the baseline with the highest sensor density.
2. The anemometers should be positioned with greatest possible distance to obstacles like beams, walls, etc., as they can significantly bias the results for volume flow rate. Such behavior was not observed for the gas concentrations.
3. The gas at the outlet area is not well mixed, resulting in high vertical and lateral gradients of gas concentrations. Hence, when deriving emissions with a direct method, the focus should be on the representative sampling of the target gas concentration. When using gas measurement systems that measure at a constant height (e.g., open path laser systems), preliminary studies for each target gas should be carried out in order to quantify the vertical concentration gradients and derive a correction factor for the height.
4. Provided that vertical composite sampling of gas concentrations is possible, a division of the opening width into units with a lateral distance of 19 m, each equipped with an anemometer positioned in the vicinity of the horizontal symmetry line, is sufficient to reach results for the direct estimation of emissions with errors less than 7%, compared to the baseline.

As the concentration gradients of the gas concentrations have a large influence on the emission estimations, the influencing factors on the gas mixing efficiency should be investigated further. Future research should therefore include variations of the gas source characteristics (e.g., uniform area source vs. different hot spots; positioned at floor level vs. positioned at animal height level) and the systematic variation of measures that potentially change the airflow pattern (e.g., different interior, partly closed opening configurations). Additionally, instead of modeling an isothermal flow, the additional modeling of buoyancy inside the barn could lead to a greater vertical mixing of gas concentrations, resulting in sensor positions less sensitive to the height. This should also be investigated further.

Direct methods have the potential to compensate for the limitations attributed to indirect methods, e.g., when emittents are located both outdoor and indoor the housing system (as in systems with outdoor walking alleys). Theoretically, the mass flow through any given system boundary can be measured with direct methods, but the measured results are highly dependent on the number of sensors. The results of this study will be of help researchers to assess the expected uncertainty associated with the resolution of sensors, when applied on the aforementioned housing systems.

## Abbreviations

The following abbreviations are used in this manuscript:

LDA	Laser Doppler Anemometer
FFID	Fast Flame Ionization Detector
AER	Air Exchange Rate
ABLWT	Atmospheric Boundary Layer Wind Tunnel
SP	Sampling Point(s)
CMV	Converged Mean Value
BL	Baseline Configuration
CFG	Configuration
